# Intestinal Epithelial-Derived Exosomes Under Cold Stimulation Promote Adipose Thermogenesis

**DOI:** 10.3390/metabo15050324

**Published:** 2025-05-14

**Authors:** Xue Han, Tiange Feng, Yaxu Yang, Ziming Zhu, Fangyu Shao, Lijun Sun, Yue Yin, Weizhen Zhang

**Affiliations:** 1 Department of Pharmacology, School of Basic Medical Sciences, State Key Laboratory of Vascular Homeostasis and Remodeling, Peking University, Beijing 100191, China; hanxuehx313@bjmu.edu.cn (X.H.); shaofangyu@bjmu.edu.cn (F.S.); 2 Department of Physiology and Pathophysiology, School of Basic Medical Sciences, State Key Laboratory of Vascular Homeostasis and Remodeling, Peking University, Beijing 100191, China1810305332@pku.edu.cn (Y.Y.); zhuziming.d@163.com (Z.Z.);

**Keywords:** intestinal epithelial cell, exosomes, adipose, thermogenesis, miRNA

## Abstract

**Background**: Whether intestinal epithelial cells can regulate distant adipose tissue remains a mystery. **Methods**: Cold-stimulated intestinal epithelial cell-derived exosomes (Cold IEC-Exo) play a pivotal role in enhancing adipose thermogenesis and metabolic homeostasis, as demonstrated in this study. **Results**: IEC-Exo can accumulate in adipose tissue. Compared with IEC-Exo derived from room temperature mice (RT IEC-Exo), Cold IEC-Exo significantly enhanced the thermogenesis of adipose. In vitro, Cold IEC-Exo directly stimulated thermogenesis in primary adipocytes by elevating oxygen consumption rate, proton leak, and fatty acid uptake, with no effect on glucose uptake. Small RNA sequencing identified miR-674-3p as a key mediator enriched in Cold IEC-Exo. miR-674-3p mimicry replicated Cold IEC-Exo effects, augmenting *Ucp1* expression, mitochondrial uncoupling, and fatty acid utilization in adipocytes. Local overexpression of miR-674-3p in BAT and sWAT via AAV in vivo enhanced thermogenesis and attenuated diet-induced glucose intolerance. **Conclusions**: These findings establish that Cold IEC-Exo, via miR-674-3p transfer, drive adipose thermogenic activation and mitigate metabolic dysfunction, highlighting their therapeutic potential in obesity-related disorders.

## 1. Introduction

Obesity, recognized as a chronic relapsing progressive disease, represents a formidable global public health challenge. China has witnessed an alarming surge in obesity prevalence, with a 2023 nationwide epidemiological study revealing that among 15.8 million adult participants, 34.8% and 14.1% met criteria for being overweight and obese, respectively [1]. These epidemiological insights underscore the imperative to prioritize obesity-related metabolic disorder management in public health strategies [2], thereby necessitating the urgent scientific exploration of safe and effective therapeutic interventions.

Enhancing systemic energy expenditure through adipose thermogenesis constitutes a pivotal anti-obesity strategy. Brown adipose tissue (BAT) and beige adipocytes, distinguished by mitochondrial uncoupling protein 1 (UCP1) expression, dissipate proton gradients across the inner mitochondrial membrane to thermogenesis via non-shivering thermogenesis. This thermogenic process drives a substantial uptake of circulating glucose and triglycerides to fuel tricarboxylic acid cycle and β-oxidation pathways, effectively lowering systemic glucose and lipid levels [3,4]. While sympathetic nervous system activation classically mediates adipose thermogenesis, emerging evidence highlights the critical role of inter-organ crosstalk in metabolic regulation. The identification of novel mediators in organ communication thus offers promising therapeutic potential for metabolic diseases.

The gastrointestinal tract, a multifunctional organ central to nutrient processing, immune homeostasis, and metabolic regulation, may orchestrate remote adipose tissue modulation through underexplored mechanisms. Current paradigms of gut–adipose axis communication emphasize neuronal pathways, enteroendocrine signals, and microbiota-derived metabolites [5,6,7]. However, the potential involvement of extracellular vesicles—particularly intestinal epithelial cell-derived exosomes (IEC-Exo)—remains elusive. Exosomes, extracellular vesicles carrying cell-specific biomolecular cargo (including proteins, lipids, and nucleic acids), exhibit functional heterogeneity contingent upon their cellular origin and physiological context [8,9]. Although intestinal exosome research predominantly focuses on autocrine/paracrine functions in mucosal immunity and epithelial repair [10,11,12,13], their endocrine capacity to regulate distant metabolic tissues remains largely uncharted.

This investigation elucidates the thermogenic regulatory role and molecular composition of cold-induced IEC-Exo, while evaluating their therapeutic potential against obesity-associated metabolic derangements. Through multi-omics characterization and functional validation, we demonstrate that IEC-Exo serve as novel inter-organ communicators capable of reprogramming adipose tissue energy metabolism.

## 2. Materials and Methods

### 2.1. Animals

Male C57BL/6J mice at 8 weeks of age were purchased from the Peking University Health Science Center. All the mice were housed in a SPF environment at the Department of Laboratory Animal Science, Peking University Health Science Center. The mice were maintained in a 12/12 h light–dark cycle with free access to water. The normal chow diet (NCD) was purchased from Beijing Keao Xieli Feed Co., Ltd., Beijing, China. The high-fat diet (HFD, D12492-Rodent Diet with 60% kcal% fat) was purchased from Research Diets, Inc., New Brunswick, NJ, USA.

### 2.2. Isolation, Identification, and Injection of IEC-Exo

Eight-week-old male C57BL/6J mice were fed with an NCD at room temperature (RT, 25 ± 2 °C) or cold stimulation (Cold, 4 ± 2 °C) for 7 days. The mice were fasted for 6 h before sacrifice. After the mice were anesthetized, the entire section of the small intestine tissue was gently excised. The feces within the intestine were removed, and the small intestine was gently turned inside out so that the mucosal layer faced outward and the muscular layer faced inward. After being successively treated with 10 mmol/L DTT and 8 mmol/L EDTA, it was shaken vigorously and the intestinal epithelial cells were collected. The IECs were placed statically in PBS for 30 min to secrete exosomes. The cell suspension was then centrifuged at 3000× *g* for 10 min. The supernatant was aspirated, passed through a 220-micron filter, and then centrifuged at 4 °C for 1 h at a centrifugal force of 100,000× *g*. The protein concentration of exosomes was detected using a BCA kit, EpiZyme Biomedical Technologu CO., Ltd., Shanghai, China. The exosomes were verified by transmission electron microscopy, the Western blotting technique, and a nanoparticle tracking analysis. IEC-Exo were injected through the tail vein at 2 μg/g body weight. The injections were conducted once a day for 7 consecutive days.

The isolated IEC-Exo was co-incubated with DiR fluorescent dye (Invitrogen, Waltham, MA, United States). Twenty-four hours after tail vein injection, the fluorescence of adipose tissue was detected using a small animal imaging system (PE IVIS Spectrum, PerkinElmer, Waltham, MA, USA).

### 2.3. In Situ Injection of BAT and sWAT

After being anesthetized, 8-week-old male C57BL/6J mice received BAT and sWAT in situ injections of 5 × 10^10^ and 10 × 10^10^ vector genomes/pad AAV9-miR-674-3p, respectively. The AAV9-miR-674-3p was constructed and packaged from Hanbio (Shanghai, China). The mice recovered 3 weeks after surgery.

### 2.4. Glucose Tolerance Test (GTT) and Insulin Tolerance Test (ITT)

HFD mice underwent GTT and ITT successively. For the GTT, the mice were fasted for 12 h. After the intraperitoneal injection of 1.5 g/kg glucose, the blood glucose levels in the tail vein blood were measured at 0, 15, 30, 60, 90, and 120 min, respectively. For the ITT, the mice were fasted for 6 h. After the intraperitoneal injection of 1.0 U/kg insulin, the blood glucose levels in the tail vein blood were measured at 0, 15, 30, 60, and 90 min, respectively.

### 2.5. Mice Body Weight and Surface Temperature

The mice were placed in an environment of 4 °C, and the anal temperature was measured at hours 0, 1, 2, 3, 4, and 5. The mice fasted but drank water freely.

After the mice were anesthetized, images of the surface temperature were taken using a FOTRIC 225 infrared camera (Shanghai FOTRIC Technologies Co., Ltd., Shanghai, China). The maximum body surface temperature of the mice was obtained by using AnalyzIR software 4.1.7. 6300.

### 2.6. Western Blotting

Freshly isolated tissues were homogenized in the RIPA buffer. The protein concentration of exosomes was detected using a BCA kit, EpiZyme Biomedical Technologu CO., Ltd., Shanghai, China. Proteins were extracted, then separated by SDS-PAGE, and transferred to the PVDF membrane. Immunoblot was performed using the antibodies, including anti-GPA33 (Ab203286, Abcam, Cambridge, United Kingdom), anti-CD9 (Ab223052, Abcam), anti-CD63 (Ab217345, Abcam), anti-TSG101 (A2216, ABclonal, Wuhan, China), and anti-β-Tubulin (M20005, Abmart, Shanghai, China).

### 2.7. Small RNA Sequencing

The purification of total exosome-derived RNA was carried out by the exoRNeasy Maxi Kit (Qiagen, Fenlo, Netherlands). A total amount of 3 μg total RNA per sample was used as an input material for the small RNA library. Sequencing libraries were generated using NEBNextRMultiplex Small RNA Library Prep Set for IlluminaR (NEB, Ipswich, MA, USA) following the manufacturer’s recommendations, and index codes were added to attribute sequences to each sample. The clustering of the index-coded samples was performed on a cBot Cluster Generation System using TruSeq SR Cluster Kit v3-cBot-HS (Illumia, San Diego, CA, USA) according to the manufacturer’s instructions. After cluster generation, the library preparations were sequenced on an Illumina Hiseq 2500/2000 platform and 50bp single-end reads were generated. The differential expression analysis of two groups was performed using the DESeq R package (3.0.3). The *p*-values were adjusted using the Benjamini and Hochberg method.

### 2.8. Transfection of microRNA Mimics in Primary Adipocytes

Primary brown preadipocytes were isolated from neonatal mice. Cells were cultured in induction medium containing insulin (1 μg/mL, I9278, Sigma-Aldrich, Saint Louis, MI, USA), T3 (1 nM, T6397, Sigma-Aldrich), IBMX (0.5 mM, I811775, MackIin, Shanghai, China), dexamethasone (1 μM, D1756, Sigma-Aldrich), and indomethacin (0.125 mM, HY-14397, MedChemExpress, Monmouth Junction, NJ, US). After induction for 2 days, the maintenance medium (1 μg/mL insulin, 1 nM T3) was transferred and cultured for 4 days. miR-674-3p mimics were transferred into adipocytes using lipo8000 (C0533, Beyotime Biotechnology, Shanghai, China). The miR-674-3p mimics were synthesized by Sangon Biotech (Shanghai, China).

### 2.9. Seahorse

Oxygen consumption rate (OCR) measurement was performed on the primary brown preadipocytes with the Agilent Seahorse XF96 (Agilent Technologies, Santa Clara, CA, USA) cellular respirometer, as previously described [14].

### 2.10. The Uptake of Fatty Acid and Glucose

The uptake of fatty acid (BODIPY, HY-W090090, MedChemExpresss, New Jersey, US) and glucose (2NBDG, N13195, Thermo Fisher Scientific Inc.) was performed on the primary brown preadipocytes, as previously described [14].

### 2.11. Quantitative Real-Time PCR

The total RNA was extracted with TRIGene Reagent (GenStar, Beijing, China). Reverse transcription was performed using the HiScript II 1st Strand cDNA Synthesis Kit (R211, Vazyme, Nanjing, China). Relative gene expression was measured with the Taq Pro Universal SYBR qPCR Master Mix (P211, Vazyme, Nanjing, China), using the Aria Real-Time PCR System (Agilent Technologies, Santa Clara, CA, USA). The primer sequences are shown in Table S1.

### 2.12. Statistics

Data were expressed as the mean ± SE. Significant differences between the two groups were evaluated with a *t*-test. A *p* value of <0.05 was considered statistically significant.

## 3. Results

### 3.1. IEC-Exo Under Cold Stimulation Promote Adipose Thermogenesis in Mice

**Figure 1:**
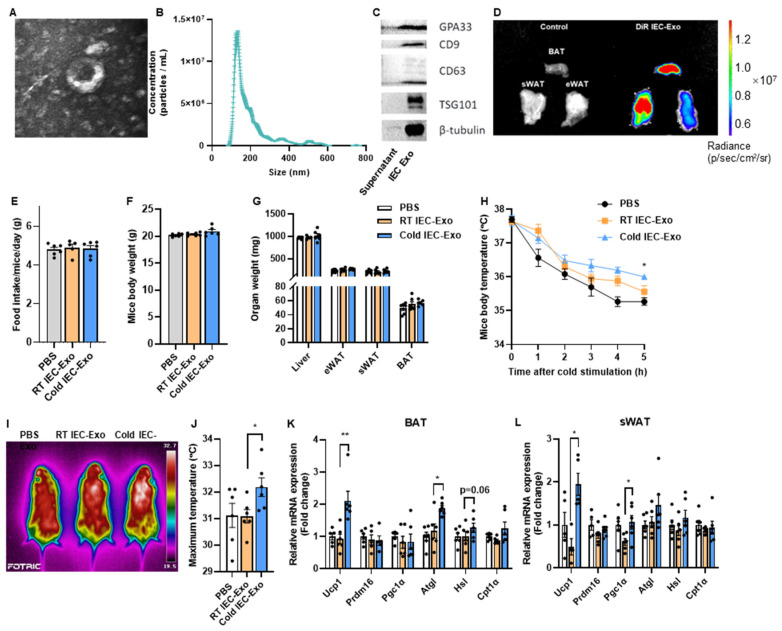
**IEC-Exo under cold stimulation promote adipose thermogenesis in mice.** (**A**) Representative transmission electron microscopy images of IEC-Exo. (**B**) Nano sight-nanoparticle tracking analysis of IEC-Exo (n = 3). (**C**) Western blotting of exosomal markers CD9, CD63, TSG101, and IEC GPA33. (**D**) Fluorescence of DiR-labeled IEC-Exo detected by IVIS Spectrum in vivo imaging system in mice. (**E**–**L**) IEC-Exo was collected from 8-week-old mice after 7 days of cold exposure at 4 °C (Cold IEC-Exo), and IEC-Exo from mice raised at room temperature (RT IEC-Exo) was used as the control. Male C57BL/6J mice fed with NCD were administered RT IEC-Exo or Cold IEC-Exo at a concentration of 2 μg/g body weight through tail vein injection. Injections were conducted once a day for 7 consecutive days (n = 6). (**E**) Food intake. (**F**) Mice body weight before sacrifice. (**G**) Organ weight. (**H**) Mice body temperature after cold stimulation. (**I**,**J**) Representative pictures of body surface temperature of mice and the statistics of the highest body surface temperature. (**K**,**L**) Relative mRNA expression of thermogenesis and fatty acid oxidation-related genes in BAT and sWAT. Each point on the column represents a separate data point. Data are presented as means ± SEM. * *p* < 0.05, ** *p* < 0.01 by T-TEST.

Small intestinal epithelial cells (IECs) from the mice were isolated through digestion with DTT and EDTA. Extracellular vesicles exhibiting a saucer-like morphology, approximately 100 nm in diameter, were successfully obtained via ultracentrifugation (Figure 1A,B). These vesicles expressed both exosomal (CD9, CD63, and TSG101) and IEC-specific (GPA33) markers (Figure 1C), confirming the successful isolation of IEC-derived exosomes (IEC-Exo). It remained unclear whether IEC-Exo could effectively accumulate in adipose tissues under physiological conditions. To address this, IEC-Exo were fluorescently labeled with DiR and administered to mice via tail vein injection. Results revealed enhanced fluorescence signals in brown adipose tissue (BAT), subcutaneous white adipose tissue (sWAT), and epididymal white adipose tissue (eWAT) (Figure 1), indicating the accumulation of IEC-Exo in adipose tissues, particularly BAT and sWAT.

Wild-type mice fed a normal chow diet (NCD) were exposed to 4 °C cold stress for 7 days, and IEC-Exo from cold-exposed mice (Cold IEC-Exo) were isolated and intravenously administered to recipient mice from their tails. The control groups received IEC-Exo from room temperature-housed NCD mice (RT IEC-Exo) or PBS. Compared to PBS-treated mice, neither RT IEC-Exo nor Cold IEC-Exo administration affected food intake, body weight, or organ weights of the liver, eWAT, sWAT, and BAT (Figure 1E–G). Importantly, Cold IEC-Exo administration improved body temperature maintenance in cold-challenged mice (Figure 1H) and significantly elevated maximum surface body temperature compared to RT IEC-Exo-treated mice (Figure 1I,J). Furthermore, Cold IEC-Exo markedly upregulated mRNA expression of Ucp1, a key thermogenic gene, in both BAT and sWAT (Figure 1K,L). Concurrently, Cold IEC-Exo enhanced the expression of fatty acid oxidation-related genes in BAT (Figure 1L), suggesting enhanced substrate availability for thermogenesis.

These findings demonstrate that IEC-Exo accumulate in adipose tissues and that IEC-Exo derived from cold-exposed mice promote thermogenic activation in adipose tissues.

### 3.2. IEC-Exo Under Cold Stimulation Promote the Thermogenesis of Primary Adipocytes

**Figure 2:**
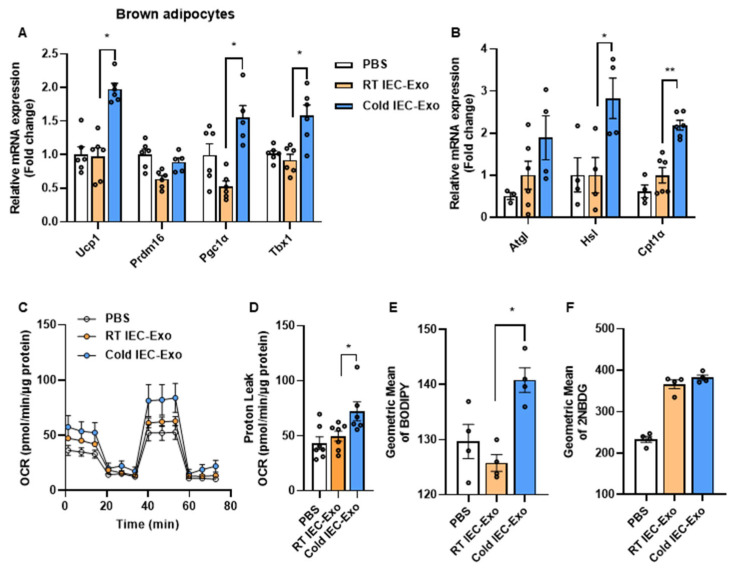
**IEC-Exo under cold stimulation promote the thermogenesis of primary adipocytes.** Relative mRNA expression of thermogenesis (**A**) and fatty acid oxidation-related (**B**) genes (n = 6). (**C**,**D**) The OCR and proton leak (n = 6). The uptake of BODIPY (**E**) and 2NBDG (**F**) (n = 4). Each point on the column represents a separate data point. Data are presented as means ± SEM. * *p* < 0.05, ** *p* < 0.01 by T-TEST.

We further investigated whether IEC-Exo under cold stimulation accelerate adipose tissue thermogenesis by directly acting on adipocytes. In primary brown adipocytes, compared to those treated with RT IEC-Exo, Cold IEC-Exo treatment significantly increased the transcriptional levels of thermogenesis- and fatty acid oxidation-related genes, including Ucp1, Pgc1α, Tbx1, Hsl, and Cpt1α (Figure 2A,B). Moreover, Cold IEC-Exo treatment markedly enhanced the oxygen consumption rate (OCR) and proton leak (an indicator of uncoupled thermogenesis) in primary brown adipocytes compared to RT IEC-Exo treatment (Figure 2C,D), indicating enhanced uncoupling capacity. Fatty acids and glucose serve as substrates for uncoupled thermogenesis in adipose tissue. Cold IEC-Exo treatment significantly increased fatty acid uptake in primary brown adipocytes but showed no significant effect on glucose uptake (Figure 2E,F). These data demonstrate that IEC-Exo under cold stimulation can directly promote fatty acid uptake in adipocytes, thereby accelerating uncoupled thermogenesis.

### 3.3. The miR-674-3p in Cold IEC-Exo Promotes the Thermogenesis of Adipocytes

**Figure 3:**
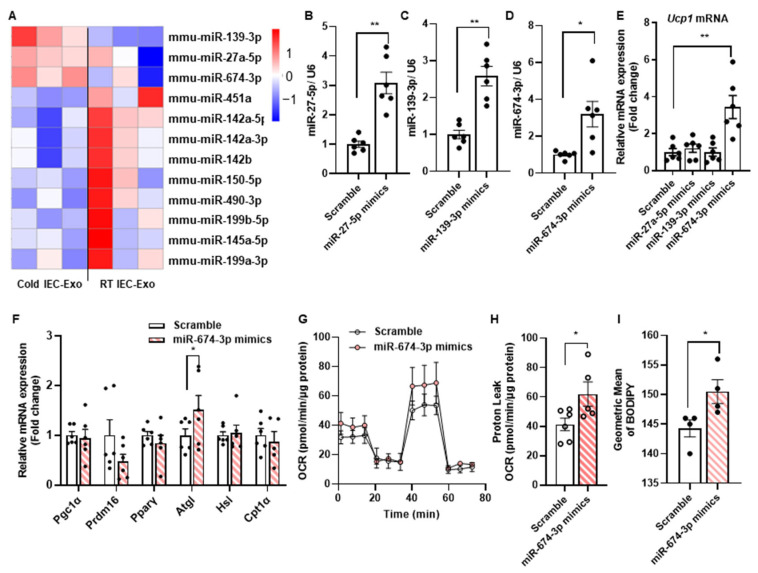
**miR-674-3p in Cold IEC-Exo promotes thermogenesis in adipocytes.** (**A**) Differentially expressed microRNAs in RT IEC-Exo and Cold IEC-Exo (n = 3). (**B**–**D**) The expression levels of *miR-27a-5p*, *miR-139-3p,* and *miR-674-3p* in primary brown adipocytes after transfection with miR-27a-5p, miR-139-3p, and miR-674-3p mimics, respectively (n = 6). (**E**) The mRNA expression of *Ucp1* in primary brown adipocytes after transfection with miR-27a-5p, miR-139-3p, and miR-674-3p mimics, respectively (n = 6). (**F**) Relative mRNA expression of thermogenesis and fatty acid oxidation-related genes in primary brown adipocytes after transfection with miR-674-3p mimics (n = 6). (**G**,**H**) The OCR and proton leak (n = 6). (**I**) The uptake of BODIPY (n = 4). Each point on the column represents a separate data point. Data are presented as means ± SEM. * *p* < 0.05, ** *p* < 0.01 by T-TEST.

Exosomes contain bioactive components within their cargo, including DNA, RNA, and proteins. It is well-established that miRNAs encapsulated in exosomes exert potent and heterogeneous functional effects. To identify the functional components involved, small RNA sequencing was performed on RT IEC-Exo and Cold IEC-Exo. Twelve differentially expressed miRNAs were identified between the two groups, with three miRNAs (miR-27a-5p, miR-139-3p, and miR-674-3p) showing increased abundance in Cold IEC-Exo (Figure 3A). Mimics of these miRNAs were transfected into primary brown adipocytes, with transfection efficiency confirmed in Figure 3B–D. miR-674-3p significantly upregulated *Ucp1* transcriptional levels in primary adipocytes, while miR-27a-5p and miR-139-3p showed no significant effect on *Ucp1* expression (Figure 3E). Furthermore, miR-674-3p markedly increased the expression of the fatty acid oxidation gene *Atgl*, elevated oxygen consumption rate (OCR) and proton leak, and enhanced fatty acid uptake (Figure 3F–I). These results suggest that miR-674-3p is a critical contributor to the Cold IEC-Exo-mediated promotion of adipose thermogenesis.

### 3.4. In Situ Overexpression of miR-674-3p in BAT and sWAT Promotes Thermogenesis in Mice

**Figure 4:**
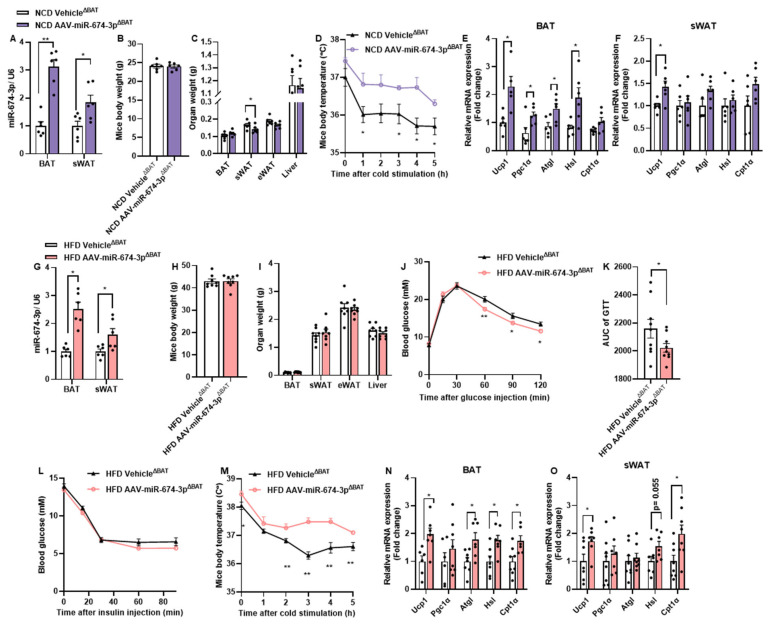
**In situ overexpression of miR-674-3p in BAT and sWAT promotes thermogenesis in mice.** (**A**–**F**) BAT and sWAT were injected in situ with AAV-miR-674-3p (n = 6). Mice were fed with NCD and experiments were conducted. The mice injected with Vehicle in situ were used as the control (n = 6). (**A**) Overexpression efficiency. (**B**) Mice body weight. (**C**) Organ weight. (**D**) Mice body temperature after cold stimulation. (**E**,**F**) Relative mRNA expression of thermogenesis and fatty acid oxidation-related genes in BAT and sWAT. (**G**–**O**) BAT and sWAT were injected in situ with AAV-miR-674-3p. Three weeks after recovery, the mice were fed with HFD for 12 weeks (n = 8). The mice injected with Vehicle in situ were used as the control (n = 8). (**G**) Overexpression efficiency. (**H**) Mice body weight. (**I**) Organ weight. (**J**,**K**) Glucose tolerance test and area under the curve (AUC). (**L**) Insulin tolerance test. (**M**) Mice body temperature after cold stimulation. (**N**,**O**) Relative mRNA expression of thermogenesis and fatty acid oxidation-related genes in BAT and sWAT. Each point on the column represents a separate data point. Data are presented as means ± SEM. * *p* < 0.05, ** *p* < 0.01 by T-TEST.

To further determine whether miR-674-3p promotes thermogenesis in BAT and sWAT in vivo, BAT and sWAT were locally injected with an adeno-associated virus carrying miR-674-3p (AAV-miR-674-3p^ΔBAT^). The levels of miR-674-3p were increased by 3.12 ± 0.62-fold in BAT and 1.85 ± 0.61-fold in sWAT, respectively (Figure 4A). The overexpression of miR-674-3p in BAT and sWAT did not affect body weight or the tissue weights of the liver, BAT, or eWAT, but reduced the sWAT weight (Figure 4B,C). Mice overexpressing miR-674-3p in BAT and sWAT exhibited attenuated body temperature decline during cold exposure (Figure 4D). Additionally, thermogenic and fatty acid oxidation-related genes were significantly upregulated in BAT and sWAT of the AAV-miR-674-3pΔBAT group (Figure 4E).

To investigate whether the miR-674-3p-mediated enhancement of adipose tissue function ameliorates obesity-related metabolic disorders, mice with BAT and sWAT locally injected with AAV-miR-674-3p were fed a high-fat diet (HFD) for 12 weeks. Following HFD, miR-674-3p remained significantly overexpressed in BAT and sWAT, though no differences in body or tissue weights were observed (Figure 4G–I). Notably, mice overexpressing miR-674-3p in BAT and sWAT showed significantly improved glucose tolerance (Figure 4J,K), while insulin tolerance remained unchanged (Figure 4L). Consistent with findings in normal chow diet (NCD)-fed mice, miR-674-3p overexpression in BAT and sWAT of HFD-fed mice markedly enhanced cold-induced thermogenesis (Figure 4M). As expected, thermogenic and fatty acid oxidation-related genes were significantly elevated in BAT and sWAT (Figure 4N,O).

These data demonstrate that the overexpression of miR-674-3p in murine BAT and sWAT robustly enhances adipose tissue thermogenesis and mitigates diet-induced glucose tolerance impairment.

## 4. Discussion

The present study unveils a previously unrecognized endocrine mechanism through which cold-induced intestinal epithelial cell-derived exosomes (IEC-Exo) enhance adipose thermogenesis. Our findings establish IEC-Exo as novel inter-organ communicators that bridge gut-derived signals to adipose metabolic reprogramming, expanding the conceptual framework of the gut–adipose axis beyond classical neuronal and endocrine pathways. By demonstrating that IEC-Exo accumulate in adipose depots and deliver thermogenic effector miRNAs such as miR-674-3p, this work provides mechanistic insights into how intestinal responses to environmental stimuli systemically regulate energy metabolism.

The thermogenic activation mediated by Cold IEC-Exo occurred independently of body weight alterations, suggesting that exosomal signaling primarily enhances energy expenditure rather than suppressing appetite or nutrient absorption. This contrasts with conventional gut-derived hormones like GLP-1, which predominantly affect satiety and glucose homeostasis [15]. The selective upregulation of UCP1-dependent uncoupling and fatty acid oxidation genes in BAT and sWAT, coupled with increased proton leak and oxygen consumption in adipocytes, highlights IEC-Exo’s capacity to directly potentiate thermogenic machinery. Notably, the preferential enhancement of fatty acid—but not glucose—uptake aligns with cold adaptation mechanisms that prioritize lipid mobilization as a primary fuel source for non-shivering thermogenesis. It is noteworthy that both the duration and temperature of cold stimulation require further detailed investigation. Furthermore, the optimal delivery routes for intestinal exosomes (e.g., oral vs. systemic administration) require systematic exploration to reconcile their physiological trafficking pathways and enhance targeting efficacy.

The physiological roles of miR-674 remain understudied. Emerging evidence suggests the dysregulation of miR-674 levels in central nervous system-related disorders. For instance, post-traumatic stress in Sprague Dawley rats upregulated miR-674-3p in both serum and the amygdala [16]. In progressive ventriculomegaly models, reduced expression of Drd1-targeting microRNAs, particularly miR-674-3p, led to elevated dopamine receptor Drd1 levels, contributing to ciliary motility defects [17]. Similarly, the altered expression of seven microRNAs, including miR-674-3p, was observed in the mesocortical neural circuitry of depression models [18]. Docetaxel combined with Fuzheng Yiliu decoction suppressed castration-resistant prostate cancer progression by modulating 10 miRNAs, including miR-674-3p [19]. Additionally, miR-674-3p was implicated in promoting stress-induced hypertension [20]. Two distinct targets of miR-674 have been identified in endothelial and hepatic injury studies. In hydrogen peroxide (H2O2)-induced senescent mouse aortic endothelial cells, the inhibition of miR-674-5p attenuated cellular senescence and oxidative stress by upregulating complement C1q/TNF-related protein 9 (CTRP9), thereby enhancing proliferation, migration, and angiogenesis [21]. In concanavalin A-induced autoimmune hepatitis models, hepatic miR-674-5p expression was significantly reduced, potentially ameliorating liver injury via the negative regulation of 5-lipoxygenase [22].

Central to this regulatory axis is miR-674-3p, identified as a key exosomal cargo responsible for adipocyte thermogenic activation. While prior studies have implicated miRNAs in inter-organ metabolic crosstalk, our multi-omics approach specifically links cold-adaptive intestinal signaling to miR-674-3p enrichment in IEC-Exo. The ability of miR-674-3p overexpression to recapitulate Cold IEC-Exo effects—enhancing thermogenic gene expression, substrate utilization, and systemic glucose tolerance—underscores its therapeutic potential. Intriguingly, miR-674-3p’s effects persisted under obesogenic conditions, improving glucose homeostasis in HFD-fed mice without altering insulin sensitivity. This suggests that miR-674-3p may target pathways distinct from canonical insulin signaling, possibly modulating mitochondrial efficiency or adipokine secretion. Future studies delineating miR-674-3p’s molecular targets in adipocytes will clarify its mechanism of action.

These findings carry translational implications for obesity management. Unlike systemic adrenergic agonists, which carry cardiovascular risks, the exosome-mediated delivery of miR-674-3p offers a tissue-specific modulation of adipose thermogenesis. Furthermore, the gut’s responsiveness to thermal stimuli suggests that dietary or pharmacological interventions targeting intestinal exosome secretion could amplify endogenous thermogenic capacity. However, challenges remain in optimizing exosome delivery and scaling production for clinical use. The absence of adverse effects on organ weights or metabolic parameters in our models supports the safety profile of IEC-Exo, though long-term studies are warranted.

This study also raises broader questions about gut–adipose communication. While cold exposure enhanced IEC-Exo’s thermogenic potency, the upstream sensors and signaling pathways governing exosomal miR-674-3p packaging remain undefined. Potential mechanisms include cold-induced sympathetic activation in the gut or microbiota-derived signals modulating IEC exosome secretion. Additionally, the contribution of non-miRNA cargo (e.g., proteins or lipids) in IEC-Exo merits exploration, as these components may synergize with miRNAs to regulate metabolism. Future research should address the translational feasibility of engineering exosomes enriched with miR-674-3p or small molecules that mimic its function, offering a novel strategy to combat the global obesity epidemic. Additionally, future studies should investigate the heterogeneity of IEC-Exo functions under diverse physiological and pathological conditions (e.g., diabetes, fasting, refeeding, etc.).

## 5. Conclusions

In conclusion, our work positions IEC-Exo as critical mediators of adaptive thermogenesis and metabolic health. By elucidating the role of miR-674-3p in gut–adipose crosstalk, this study advances the development of exosome-based therapies for obesity and its comorbidities.

## Data Availability

All other data that support the findings of this study are available from the corresponding author upon reasonable request.

## Supplementary Materials

The following supporting information can be downloaded at: https://www.mdpi.com/article/10.3390/metabo15050324/s1, Table S1: Primer sequences.
