# Supplementary material for: Intestinal Epithelial-Derived Exosomes Under Cold Stimulation Promote Adipose Thermogenesis
Source: Metabolites. 2025 May 14;15(5):324. doi: 10.3390/metabo15050324 (PMC12113151; doi:10.3390/metabo15050324)
Supplement: Supplementary file 1 [file metabolites-15-00324-s001.zip › metabolites-3631625-supplementary.pdf]

Table S1. Primer sequences

| | Forward | Reverse |
|---|---|---|
| *Ucp1* | GTGAACCCGACAACTTCCGAA | TGCCAGGCAAGCTGAAACTC |
| *Prdm16* | TGCTGACGGATACAGAGGTGT | CCACGCAGAACTTCTCGCTAC |
| *Pgc1α* | TATGGAGTGACATAGAGTGTGCT | GTCGCTACACCACTTCAATCC |
| *Atgl* | ATGTTCCCGAGGGAGACCAA | GAGGCTCCGTAGATGTGAGTG |
| *Hsl* | GATTTACGCACGATGACACAGT | ACCTGCAAAGACATTAGACAGC |
| *Cpt1α* | TGGCATCATCACTGGTGTGTT | GTCTAGGGTCCGATTGATCTTTG |
| *Tbx1* | GTCAAGGCTCCGGTGAAGAAG | GCTGATTGAACTCGTCCCACA |
| *Pparγ* | GCATGGTGCCTTCGCTGA | TGGCATCTCTGTGTCAACCATG |
